# Mind the Depth: Visual Perception of Shapes Is Better in Peripersonal Space

**DOI:** 10.1177/0956797618795679

**Published:** 2018-10-04

**Authors:** Elvio Blini, Clément Desoche, Romeo Salemme, Alexandre Kabil, Fadila Hadj-Bouziane, Alessandro Farnè

**Affiliations:** 1 Integrative Multisensory Perception Action & Cognition Team (ImpAct), INSERM U1028, CNRS UMR5292, Lyon Neuroscience Research Center (CRNL), Lyon, France; 2 University of Lyon 1; 3 Hospices Civils de Lyon, Neuro-Immersion Platform, Lyon, France

**Keywords:** peripersonal space, depth, multisensory integration, perception, visual streams

## Abstract

Closer objects are invariably perceived as bigger than farther ones and are therefore easier to detect and discriminate. This is so deeply grounded in our daily experience that no question has been raised as to whether the advantage for near objects depends on other features (e.g., depth itself). In a series of five experiments (*N* = 114), we exploited immersive virtual environments and visual illusions (i.e., Ponzo) to probe humans’ perceptual abilities in depth and, specifically, in the space closely surrounding our body, termed peripersonal space. We reversed the natural distance scaling of size in favor of the farther object, which thus appeared bigger, to demonstrate a persistent shape-discrimination advantage for close objects. Psychophysical modeling further suggested a sigmoidal trend for this benefit, mirroring that found for multisensory estimates of peripersonal space. We argue that depth is a fundamental, yet overlooked, dimension of human perception and that future studies in vision and perception should be depth aware.

Closer objects are invariably perceived as bigger than farther ones and are therefore easier to detect and discriminate. This is so intuitively clear that, to date, no question has been raised as to whether the advantage for near objects depends on their size (larger) or distance (smaller). Objects located close to our body (in peripersonal space, or PPS) may benefit from processing by specialized mechanisms: Visual responses in multisensory regions are specifically tuned to close stimuli (Fogassi et al., 1996; Rizzolatti, Matelli, & Pavesi, 1983) in both human brains (Brozzoli, Gentile, Petkova, & Ehrsson, 2011; Làdavas, 2002) and nonhuman primate brains (Fogassi et al., 1996; Graziano & Cooke, 2006; Rizzolatti, Fadiga, Fogassi, & Gallese, 1997). The notion that, as a consequence, perceptual abilities would not be the same across the three orthogonal axes stemming from the body is not new (de Gonzaga Gawryszewski, Riggio, Rizzolatti, & Umiltá, 1987). Yet the majority of perception studies have so far neglected the sagittal, near-to-far dimension (van der Stoep, Serino, Farnè, Di Luca, & Spence, 2016).

One possibility is that such specialization for PPS might reflect reactive or defensive behavior in response to potentially harming or noxious stimuli (Graziano & Cooke, 2006; Makin, Holmes, Brozzoli, Rossetti, & Farnè, 2009; Sambo, Forster, Williams, & Iannetti, 2012). The multimodal—visual, tactile, and auditory—responsiveness of neurons in areas such as the ventral intraparietal area (Graziano & Cooke, 2006) would be optimal for the coding and maintaining of a safety boundary around the body (Canzoneri, Magosso, & Serino, 2012; Ferri, Tajadura-Jiménez, Väljamäe, Vastano, & Costantini, 2015; Serino, Canzoneri, & Avenanti, 2011; Teneggi, Canzoneri, di Pellegrino, & Serino, 2013), possibly coordinating automatic defensive behavior whenever necessary (Graziano & Cooke, 2006). Furthermore, objects lying in proximity to the body might more often be candidates for manipulation, and thus, the enhanced PPS processing might reflect an attempt to maximize prehension efficiency or any voluntary action toward these objects (Brozzoli, Ehrsson, & Farnè, 2014; Brozzoli, Gentile, & Ehrsson, 2012). The functional linkage between PPS and actions, supported by neurophysiological and anatomical evidence from primate work (for a review, see Makin, Holmes, Brozzoli, & Farnè, 2012), prompted the idea that visual processing in PPS would mainly rely on the dorsal visual stream, optimized for action, whereas visual processing beyond it, in extrapersonal space (EPS), would mainly rely on the ventral stream, optimized for perception (Milner & Goodale, 2008; Previc, 1990).

This presupposed division of labor indicates that object detection would be more efficient for stimuli appearing close to the body, in light of the recruitment of parietal networks tapping on magnocellular processing (Milner & Goodale, 2008). This has been generally confirmed (de Gonzaga Gawryszewski et al., 1987; Plewan & Rinkenauer, 2017). In contrast, object discrimination would be more efficient for stimuli appearing far from the body, in light of the enhanced reliance on a ventral, parvocellular pathway (Goodale & Milner, 1992). Because retinal size scales with physical distance, it appears sound to ascribe perceptual processing in EPS to a subset of neurons that present higher spatial resolution (Goodale & Milner, 1992). However, to be appropriate, automatic defensive reactions to objects in the PPS require the brain to quickly discern whether objects are indeed harmful (e.g., bees) or not (e.g., ladybugs). Similarly, voluntary appetitive actions on objects in the PPS would require discriminating between the shapes of the objects. We therefore hypothesized that object discrimination may also benefit from PPS processing. To date, whether object-discrimination abilities are superior in PPS or EPS remains unanswered.

Here, we capitalized on immersive virtual environments that, compared with 2-D settings, provide clear depth percepts. We presented geometric shapes either close (50 cm, within PPS) or far (300 cm, in EPS) from healthy volunteers engaged in a shape-discrimination task (depth was thus irrelevant and orthogonal to the task at hand). Our aims were (a) to compare discrimination abilities in PPS and EPS when retinal-size scaling is artificially teased apart, (b) to explore the determinants of any depth-related difference (i.e., perspective vs. binocular cues), and (c) to model the spatial distribution of discrimination abilities in depth.

In the first experiment, we found that discrimination abilities are superior for stimuli presented in PPS compared with stimuli presented outside PPS, despite far stimuli having the same retinal size (thus looking bigger). In Experiment 2, we found that this advantage persists in a 2-D setting exploiting perspective cues (i.e., in the context of the Ponzo illusion), thus showing that binocular depth cues are not necessary in order to highlight an advantage for PPS. Experiment 3 further replicated results from the first experiment, ruling out a potential confound related to upper/lower visual field covariance with depth—that is, stimuli were presented at the same height (at fixation). In Experiment 4, retinal size was naturally scaled as a function of distance, allowing us to estimate the typical strength of the PPS advantage in more ecological settings. Finally, in Experiment 5, we presented shapes at six different distances and found that benefits over performance follow a sigmoidal trend, closely mirroring that found in studies using multisensory integration to probe PPS boundaries (Canzoneri et al., 2012; Ferri et al., 2015; Teneggi et al., 2013).

## Method

### Participants

Participants were healthy volunteers who were enrolled in the study after we obtained informed written consent. They were all students of the University Claude Bernard of Lyon, were recruited through web advertising, and were paid for their participation. None of the participants had a history of neurologic or psychiatric disorders, and the vision of all participants was normal or corrected to normal.

We had no prior beliefs or pilot data to estimate a realistic effect size. We recruited 20 participants for Experiment 1 because this number reflects the average sample size in similar PPS studies. Once results were obtained, a power analysis (paired-samples *t* test, Cohen’s *d* = 0.6, α = .05, one-tailed) indicated a minimum of 19 participants to reach a power of .8 (the effect size from Experiment 1 was computed as if reflecting a between-participants design, and thus, this power analysis revealed itself to be conservative). About 20 participants were thus enrolled for each of the following experiments, except for Experiment 2, which was performed concurrently with a parallel experiment that required a larger sample size. The recruitment was made independently for each of the five experiments, but recruitments for Experiments 3 and 4 were made in parallel, and a few participants completed both experiments; those participants always performed Experiment 3 before Experiment 4. In no case were optional stopping procedures applied; the experiments ended either because the prespecified number of participants was reached or (in Experiment 2) because other experiments running in parallel stopped as well. Thus, the significance of the results was never considered as a criterion to stop or continue data collection. A summary of demographic information for each experiment is reported in Table 1. The study followed the Declaration of Helsinki standards and was approved by the Institut National de la Santé et de la Recherche Médicale (INSERM) Ethics Committee (IRB00003888, No. 16-341).

**Table 1.** Demographic Information for the Five Experiments

Experiment	Sample size	Left-handed (*n*)	Age (years)	
			*M*	*SD*
1	20 (10 female)	2	23.4	3.13
2	32 (16 female)	2	21.8	2.52
3	21 (10 female)	6	23.9	2.06
4	21 (11 female)	6	24.6	2.58
5	20 (10 female)	0	24	3.94

### Materials and apparatus

We adapted the task designed by O’Connor, Meade, Carter, Rossiter, and Hester (2014), which was originally employed to test spatial sensitivity to reward, reported to be reduced in far relative to near space. In Experiments 1, 3, 4, and 5, participants wore a virtual-reality headset (Oculus Rift; https://www.oculus.com). The experiments were implemented within Unity (Version 5.1.2; Unity Technologies, San Francisco, CA) and Oculus Runtime (Version 0.6; Facebook Technologies Ireland, Dublin, Ireland) software, which were used to create the virtual environment, display experimental stimuli on the head-mounted display, and record participants’ responses. The experiments were run on a computer with an Intel Core i7 processor, AMD FirePro M6000 graphics card, and Windows 7 operating system. The scene was rendered in Oculus Rift DK2 software, with a resolution of 960 × 1,080 per eye, a frequency of 75 Hz, and a field of view equal to 106°.

In Experiment 2, participants faced a 15-in. screen at a distance of approximately 57 cm. The open-source software OpenSesame (http://osdoc.cogsci.nl/) was used to display experimental stimuli and record participants’ responses. Stimuli were obtained with professional designing software (SolidWorks; Dassault Systèmes, Waltham, MA). The rendering of an empty room was designed to introduce depth cues by exploiting a Ponzo-like illusion. A very similar empty room was also created and presented as a virtual environment in Experiments 1, 3, 4, and 5.

Across all experiments, we obtained different distance conditions by presenting red, green, or blue shapes (cubes or spheres) at different positions. Shapes were presented close to (50 cm) or far from (300 cm) the observer in the virtual environment. Note that only close shapes were within reachable distance. In Experiment 2, shapes were presented in either the bottom or upper part of the grid, providing 2-D perspective cues; thus, shapes presented in the bottom of the grid were illusorily perceived to be closer to participants. Finally, in Experiment 5, shapes were presented at six equidistant points, ranging from 50 to 300 cm.

The retinal size of the shapes (≈14° of visual angle in the 3-D experiments, ≈2.2° in the 2-D experiment) was kept constant across distances and shapes, resulting in the more distant shapes being larger (Experiments 1 and 3) or appearing illusorily larger because of the perspective (Experiment 2). In Experiments 4 and 5, retinal size was naturally scaled: Farther shapes had the same real dimensions as closer ones, and thus retinal size was smaller.

**Fig. 1.**
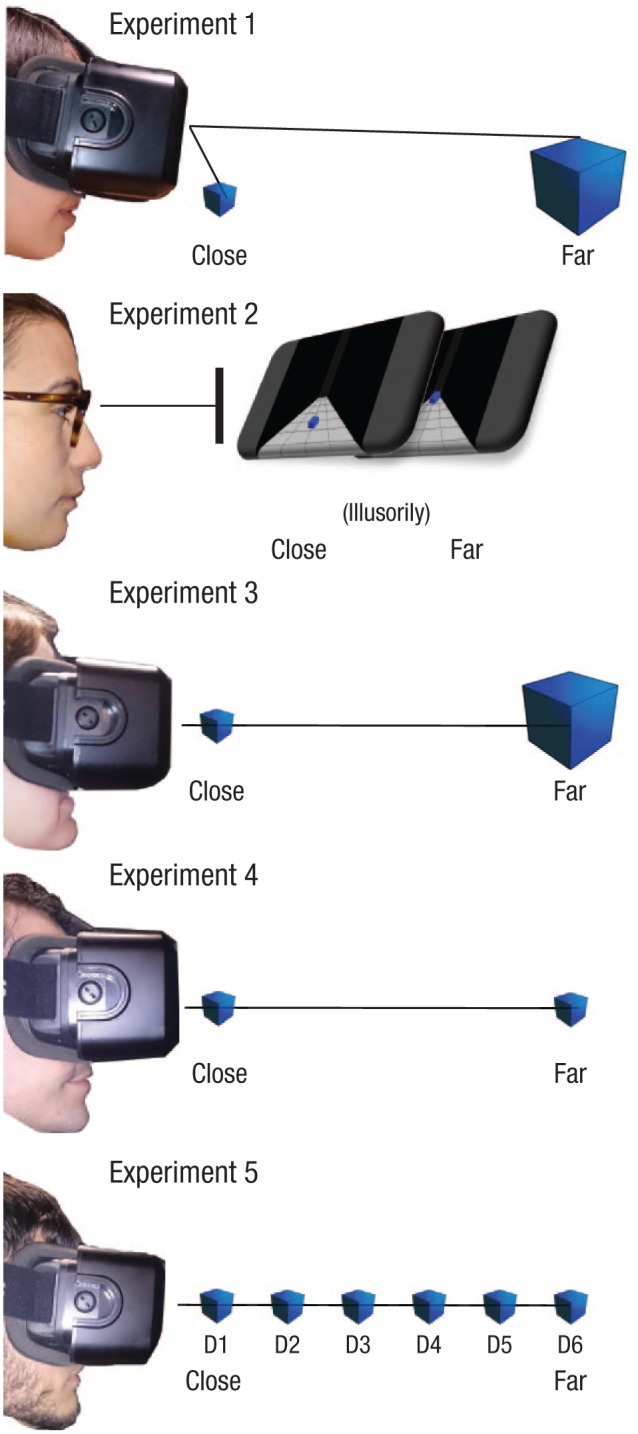
The main features of each experiment. Experiment 1 exploited a 3-D virtual-reality setting. Shapes were presented close to (50 cm) or far away from (300 cm) participants, below the fixation cross; this resulted in close shapes always being perceived to be lower than farther ones. Retinal size was kept constant. The proprioceptive input coming from the position of the hand was manipulated to be close to or far from the closer shape. Experiment 2 exploited a Ponzo illusion in a 2-D display. Shapes were presented in the lower (close) or upper (far) visual field. Retinal size was kept constant. Experiment 3 exploited a 3-D virtual-reality setting. Unlike in Experiment 1, shapes were presented at the fixation level, and their position on the transverse axis and retinal size were kept constant. Experiment 4 exploited a 3-D virtual-reality setting. Shapes were presented at the fixation level, and their position on the transverse axis was kept constant, but retinal size varied, being naturally scaled as a function of distance. Experiment 5 exploited a 3-D virtual-reality setting. Shapes were presented at the fixation level and at six different distances (50, 100, 150, 200, 250, and 300 cm, labeled D1 to D6 here). Retinal size was scaled as a function of distance.

In Experiment 1, closer shapes appeared in the bottom part of the visual field (below the fixation cross), and farther ones appeared in the upper visual field. In Experiment 2, the Ponzo-like illusion display imposed the same up-down arrangement by design (to allow a proper depth illusion). We ruled out this potential confound in Experiments 3, 4, and 5, in which all shapes were presented at the same height as the fixation cross. For all experiments, a further rendering included a cross, which was used as a fixation point across all trials. The position of the cross was midway between close and distant shapes (175 cm). Participants provided responses to object shape by means of keyboard presses (B and N keys on a standard QWERTY keyboard) using the index and middle fingers of their dominant hand. Figure 1 depicts the main features manipulated in each experiment.

### Procedure

Participants sat in a dark, quiet room, with their head restrained by a chin rest. Each trial was composed of a first fixation phase (500 ms), followed by the presentation of a stimulus randomly chosen among the combination of shape (cube or sphere), color (red, green, blue), and distance (close or far). Stimuli were presented up to a maximum of 750 ms and were replaced by feedback (text presented for 1,000 ms) as soon as a response was provided. Participants were told that responses slower than 500 ms and faster than 100 ms would be discarded, to discourage anticipations or slow responses; they were asked to respond as quickly and accurately as possible using their index finger to indicate a cube and middle finger for a sphere if responding with their right-dominant hand (the opposite finger assignment was given to left-handers). In our design, distance was therefore irrelevant to the task and orthogonal to the response.

All participants underwent a brief 24-trial practice block before starting the experiment, which consisted of another four blocks of trials. In Experiments 2 to 4, there were 60 trials each (240 trials overall). In Experiment 5, each of the four blocks was composed of 108 trials (432 overall). In Experiment 1, the whole procedure was repeated twice (i.e., four blocks of 60 trials each × 2), with a postural manipulation defining the two sessions: We asked participants to place their unseen nondominant hand in two different positions, namely, close to the chin rest (about 10 cm from their body) or farther away (roughly 50 cm from their body and therefore close to where the near virtual shape was presented). The order for hand position was counterbalanced across participants. We dropped this factor in the subsequent experiments because it had no effect on performance. The hand was therefore kept at about 10 cm from the body in the subsequent experiments.

About halfway through and after each experiment exploiting virtual reality (i.e., Experiments 1, 3, 4, and 5), we asked participants whether they had perceived two different distances and then to provide an approximate estimation for each of them. We used estimated distances given after the experiment (to allow adjustments after the initial response) to check for the presence of an effective depth perception. Several authors (for a review, see Renner, Velichkovsky, & Helmert, 2013) have found that explicit distance judgments are often underestimated by up to about 75% of the intended depths. Although here we probed the effect of distance implicitly, as it was task irrelevant, we use the labels “close” and “far” throughout the text and refrain from linearly mapping unities of the virtual environment to real distances.

### Analyses

The raw data, the full analysis pipeline, and additional graphical depictions for all experiments can be found in the Supplemental Material available online. Data, excluding practice trials, were analyzed with the open-source software R (R Core Team, 2008). Accuracy and response times (the latter for responses that were both accurate and given within the window of 100–500 ms) were analyzed through mixed-effects multiple regression models (Baayen, Davidson, & Bates, 2008). A great advantage of mixed-effects models is that they are based on single-trial data (rather than on averaged data), they do not assume independence among observations, and the model-fitting procedure takes into account the covariance structure of the data, including random effects (i.e., individual variability). Models had a logistic link function, appropriate for binary variables, when assessing accuracy.

As a first step, we defined a model containing the random effects. Linear mixed models generalize best when one includes the most complex random structure that does not prevent model convergence (Matuschek, Kliegl, Vasishth, Baayen, & Bates, 2017). Random effects were introduced sequentially, and their effect on model fit was assessed using likelihood tests (i.e., we compared the residuals of each model and chose the one with significantly lower deviance as assessed by a chi-square test). A random intercept for participant was included in all models. We then tested the contribution of random slopes for distance, hand position (Experiment 1 only), color of the presented shape, and shape. The latter variable (i.e., the presented shape, cube, or sphere) also indicates the response effector (i.e., index or middle finger), as contingencies were blocked for each participant, and thus indexes differences in discrimination performance of cubes compared with spheres and of responses with one effector over another. Finally, we also tested *n*-way interactions of random slopes that were previously retained in the models.

The models with the final random-effects structure were then used to evaluate the role of fixed effects. We used a stepwise Type 2 approach and likelihood tests to assess whether the improvements in model fit were statistically significant. Parametric bootstrapping was used to obtain 95% confidence intervals (CIs) for the beta coefficients and thus to evaluate the distribution of estimated mean differences between the levels of a factor. Additional analyses (e.g., analyses of variance, *t* tests) were also performed and are reported in the Supplemental Material in the Robustness Checks sections. All the robustness checks fully confirmed the results from the main inferential approach.

In Experiment 5, we explicitly required models to have only a random slope and fixed effect for distance. This allows obtaining, for each participant, estimates of the performance that are weighted by the random effects themselves and by the participant-specific and group-specific variances (e.g., noise; Baayen et al., 2008). We used such random slopes as dependent variables and evaluated which curve (among linear, logarithmic, exponential, and sigmoidal) best described their relationship with depth (the independent variable). The models’ formulas are reported in Table 2. Nonlinear least-squares estimations were obtained using the *nls()* function in R, and goodness of fit was evaluated by means of both root-mean-square error (RMSE) and the Akaike information criterion (AIC). The first is a measure of dispersion of residuals, whereas the latter is best used for model comparison and accounts for both goodness of fit and complexity of the models. Because the fourth model (sigmoidal) included two more parameters, the AIC introduced a more severe penalization aimed at decreasing the chances of overfitting noise.

**Table 2.** Models Contrasted in Experiment 5

Curve	Equation
Linear	y=a+b∗x
Logarithmic	y=a+b∗log(x)
Exponential	y=a+b∗exp(x/100)
Sigmoidal	$y=a+\frac{b-a}{1+\exp\left(c*(x-d)\right)}$

## Results

### Experiment 1

#### Preliminary selection of random effects

The null models included random slopes for hand position and shape when accuracy was assessed. The best matrix of random effects for response times was more complex because it included a further random slope for distance and the Distance × Shape interaction. We used these specifications to test the contribution of fixed effects through a chi-square test for goodness of fit.

#### Accuracy

Neither distance, χ^2^(1, *N* = 20) = 0.04, *p* = .84, nor hand position, χ^2^(1, *N* = 20) = 2.7, *p* = .10, improved model fit. In addition, fit did not improve when the Distance × Hand Position interaction (and main effects) was tested against the model including only the two main effects, χ^2^(1, *N* = 20) = 1, *p* = .315. Thus, none of our manipulations, or the interaction, had substantial effects on the odds of producing an accurate response. Indeed, accuracy was quite high for both the close position (*M* = 89%, *SD* = 7.88%) and the far position (*M* = 89%, *SD* = 8.47%).

#### Response times

**Fig. 2.**
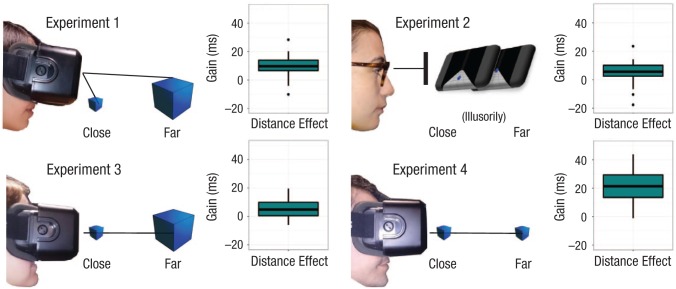
Results from Experiments 1 to 4: box-and-whisker plots depicting the mean gain in response time as a function of distance (interindividual variability of the peripersonal-space advantage, calculated by subtracting response times to close objects from response times to far objects, in ms). In each plot, the vertical length of the box represents the interquartile range, the thick horizontal line represents the median, and the whiskers indicate the full range of values. Dots outside the whiskers represent values exceeding 1.5 times the interquartile range.

Response times were considered for accurate and fast (< 500 ms) responses only, and 82.5% of the observations met this prespecified criterion (close: *M* = 83.3%, *SD* = 10.5%; far: *M* = 81.75%, *SD* = 10.3%). Response times markedly differed across viewing distances, χ^2^(1, *N* = 20) = 17.52, *p* < .001, Cohen’s *d* = 1.17, 95% CI = [0.48, 1.86] (see Fig. 2), participants being faster in categorization when shapes appeared close (*M* = 365.8 ms, *SD* = 15.06) rather than far (*M* = 375.4 ms, *SD* = 15.76), β = 13.24, *SE* = 1.65, 95% CI = [9.5, 16.5]. We observed no main effect of hand position, χ^2^(1, *N* = 20) = 1.05, *p* = .30, Cohen’s *d* = −0.16, 95% CI = [−0.8, 0.48], or the Distance × Hand Position interaction, χ^2^(1, *N* = 20) = 0.1, *p* = .753. In other words, results point toward the presence of a clear advantage for stimuli appearing in PPS, whereas the proprioceptive information coming from the hands exerted no main or modulatory effects.

Results were confirmed by a two-way analysis of variance, which yielded a main effect of distance, *F*(1, 19) = 27.34, *p* < .001, η_*p*_^2^ = .59, but no effects of hand position, *F*(1, 19) = 0.54, *p* = .47, η_*p*_^2^ = .028. The interaction between the two was not significant, *F*(1, 19) = 0.03, *p* = .858, η_*p*_^2^ = .002. Far shapes were, on average, discriminated more slowly than close shapes (mean difference = 9.62 ms, 95% CI = [5.77, 13.47]).

#### Discussion

In this experiment, visual stimuli were presented in an immersive 3-D setting using a virtual-reality headset. Despite the retinal size of different shapes being kept constant, and the farther ones being—and appearing—much bigger, we observed a response advantage to objects presented in PPS, even if they looked smaller (see Fig. 2). Whether participants placed their unseen nondominant hand close to, or far from, the more proximal virtual shape had no role in modulating the distance effect. This suggests that when only proprioception is available, the shape-discrimination advantage in PPS is not hand centered.

### Experiment 2

#### Preliminary selection of random effects

No random slope improved model fit when accuracy was assessed; random slopes for distance and shape, together with their interaction, were selected when the role of fixed effects over response times was assessed.

#### Accuracy

Accuracy was high for both the close condition (*M* = 93.8%, *SD* = 5.3%) and far condition (*M* = 94.4%, *SD* = 4%). Distance, χ^2^(1, *N* = 32) = 1.52, *p* = .217, did not improve model fit. Thus, it had no effect on the odds of producing an accurate response.

#### Response times

Response times were considered for accurate and fast (< 500 ms) responses only, and 83% of the observations met this prespecified criterion (close: *M* = 82.8%, *SD* = 14%; far: *M* = 83.3%, *SD* = 11.4%). Adding distance as a main effect improved model fit, χ^2^(1, *N* = 32) = 6.11, *p* = .0134, Cohen’s *d* = 0.68, 95% CI = [0.17, 1.20]. The response times were faster for close objects (*M* = 391.1 ms, *SD* = 16.9) compared with far objects (*M* = 396.3 ms, *SD* = 17.6), β = 4.25, *SE* = 1.34, 95% CI = [1.32, 7.09]. Results, depicted in Figure 2, were confirmed by a two-tailed *t* test for dependent samples, *t*(31) = 3.86, *p* < .001. Far shapes were, on average, discriminated more slowly than close shapes (mean difference = 5.26 ms, 95% CI = [2.48, 8.03]).

#### Discussion

The perception of depth allowed by the virtual-reality headset is due to both binocular cues (ocular disparity) and related ocular vergence, as well as to perspective cues. To isolate the role played by perspective cues in this experiment, we presented stimuli on a 2-D screen, using the rendering of an empty room as a background (Ponzo illusion). We still observed the advantage for shapes that appeared—illusorily—closer to participants, indicating that perspective cues alone are sufficient for the PPS advantage to emerge (see Fig. 2).

### Experiment 3

#### Preliminary selection of random effects

No random slope improved model fit when accuracy was assessed; a random slope for shape was instead introduced when the role of fixed effects over response times was assessed.

#### Accuracy

Accuracy was high for both the close position (*M* = 92.9%, *SD* = 4.4%) and far position (*M* = 93%, *SD* = 4.6%). Distance, χ^2^(1, *N* = 21) = 0.01, *p* = .911, did not improve model fit. Thus, it had no effect on the odds of producing an accurate response.

#### Response times

Response times were considered for accurate and fast (< 500 ms) responses only, and 85.8% of the observations met this prespecified criterion (close: *M* = 86%, *SD* = 9%; far: *M* = 85.6%, *SD* = 10.5%). Adding distance as a main effect improved model fit, χ^2^(1, *N* = 21) = 10.17, *p* = .001, Cohen’s *d* = 0.72, 95% CI = [0.08, 1.37]. The response times were faster for close objects (*M* = 371.6 ms, *SD* = 18.33) compared with far objects (*M* = 376.7 ms, *SD* = 20.0), β = 5.0, *SE* = 1.57, 95% CI = [1.92, 8.05]. Results, depicted in Figure 2, were confirmed by a two-tailed *t* test for dependent samples, *t*(20) = 3.34, *p* = .003. Far shapes were, on average, discriminated more slowly than close shapes (mean difference = 5.15 ms, 95% CI = [1.93, 8.37]).

#### Discussion

In both Experiments 1 and 2, depth covaried with the height of stimuli in the visual field, such as in ecological situations in which closer objects usually appear in the lower hemifield (Previc, 1990). Nevertheless, even when shapes were presented along the same gaze line in Experiment 3 (and hence, such a potential confound was ruled out), the advantage for stimuli in PPS was confirmed (see Fig. 2).

### Experiment 4

#### Preliminary selection of random effects

The random slope for distance improved model fit when accuracy was assessed; a further random slope for shape, together with its interaction term with distance, was included when response times were assessed. We used these specifications to test the contribution of fixed effects through a chi-square test for goodness of fit.

#### Accuracy

Accuracy was high for the close position (*M* = 89.2%, *SD* = 5.6%) and slightly, but not significantly, lower for the far position (*M* = 86.6%, *SD* = 8%). Distance, χ^2^(1, *N* = 21) = 3.38, *p* = .066, did not improve model fit.

#### Response times

Response times were considered for accurate and fast (< 500 ms) responses only, and 78.4% of the observations met this prespecified criterion (close: *M* = 82.8%, *SD* = 13.2%; far: *M* = 74%, *SD* = 16.5%). Adding distance as a main effect improved model fit, χ^2^(1, *N* = 21) = 28.9, *p* < .001, Cohen’s *d* = 1.71, 95% CI = [0.99, 2.44]. Response times were faster for close objects (*M* = 375.8 ms, *SD* = 21) compared with far objects (*M* = 397.9 ms, *SD* = 18.6), β = 20.76, *SE* = 2.33, 95% CI = [16.01, 25.8]. Results, depicted in Figure 2, were confirmed by a two-tailed *t* test for dependent samples, *t*(20) = 7.86, *p* < .001. Far shapes were, on average, discriminated more slowly than close shapes (mean difference = 22.05 ms, 95% CI = [16.2, 27.91]).

#### Discussion

This experiment, performed in ecologically veridical conditions in which farther objects appeared smaller than closer ones, demonstrates that the natural distance scaling of size substantially enhances the PPS advantage (see Fig. 2). As in the previous experiments, results cannot be ascribed to speed/accuracy trade-offs.

### Experiment 5

#### Psychophysical modeling

Random slopes (for both accuracy and response times) were fitted for each participant and for the group average to four different equations (see Table 2). At the group level, a sigmoidal trend emerged when we assessed both accuracy (sigmoidal AIC = −8.5; exponential AIC = −7.94) and response times (sigmoidal AIC = 36.44; exponential AIC = 36.95; linear AIC = 37.37). At the individual participant level, the sigmoidal trend obtained the best performance for all participants and for both response times and accuracy when using the RMSE as an index of goodness of fit. The AIC was less conclusive. When response times were assessed, the AIC still favored the sigmoidal trend for 11 participants out of 20, but for the remaining participants, the exponential curve was preferred. The results when fitting accuracy were similar, but the exponential curve was favored for 11 participants; of the remaining participants, 8 showed a sigmoidal trend, and only 1 showed a logarithmic trend. Results are summarized in Table 3.

**Table 3.** Results From Experiment 5

Measure and curve	Root-mean-square error (RMSE)	Akaike information criterion (AIC)
Accuracy		
Linear	0.13 (*n* = 0)	−1.72 (*n* = 0)
Logarithmic	0.21 (*n* = 0)	4.28 (*n* = 1)
Exponential	0.08 (*n* = 0)	−7.94 (*n* = 11)
Sigmoidal	0.05 (*n* = 20)	−8.5 (*n* = 8)
Response time		
Linear	3.3 (*n* = 0)	37.37 (*n* = 0)
Logarithmic	5.6 (*n* = 0)	43.69 (*n* = 0)
Exponential	3.19 (*n* = 0)	36.95 (*n* = 9)
Sigmoidal	2.19 (*n* = 20)	36.44 (*n* = 11)

Note: The table gives values for group means, fitted with the relative equations. The number of participants who favored each model is reported in parentheses.

#### Discussion

**Fig. 3.**
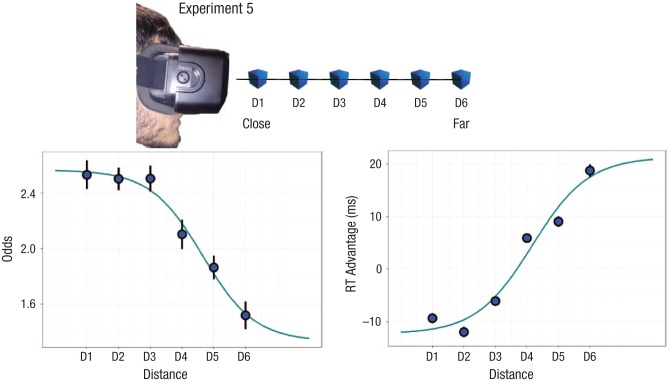
Results from Experiment 5, in which we presented shapes at six different depths. Group-wise predicted sigmoidal curves are shown for mean accuracy (left panel) and mean response time (RT) advantage (right panel) as a function of distance (labeled here from D1, close, to D6, far). Error bars show standard errors of the mean. The *y*-axes refer to the odds of providing a correct response (accuracy) and the relative RT advantage observed with respect to participant-specific mean performance.

To model the spatial tuning of shape discrimination as a function of depth in Experiment 5, we presented shapes, not corrected for retinal size, at six different, equidistant points ranging from 50 cm to 300 cm. The fit to empirical data for several theoretical curves (sigmoidal, linear, logarithmic, and exponential) was then contrasted. A sigmoidal trend emerged at the group level when we assessed both accuracy and response times (see Fig. 3). Thus, the PPS advantage follows a sigmoidal trend, similar to what is commonly observed in studies using multisensory integration paradigms to assess the PPS boundary (e.g., Canzoneri et al., 2012; Ferri et al., 2015; Teneggi et al., 2013), except that here, only the visual modality was involved.

## General Discussion

Throughout the same discrimination task, the features of different visual shapes were progressively stripped of important depth cues: (a) retinal-size differences in Experiments 1 and 3, (b) binocular cues as well as convergent and divergent eye movements in Experiment 2, and (c) upper/lower visual field covariance with depth in Experiments 3 and 4. Despite such drastic reductions, which ultimately left the mere illusion of depth perception, participants remained faster in discriminating close shapes in the absence of speed/accuracy trade-offs. This firmly indicates that close space is, per se, special and benefits from enhanced perceptual processing, even under extremely disadvantageous conditions (i.e., closer shapes being clearly smaller). It would be tempting to ascribe the PPS advantage in one of the most fundamental perceptual properties of objects such as shape discrimination to a specialized neural system. However, the ventral/dorsal dichotomy alone, although extensively supported by physiological and neuropsychological studies, cannot readily account for the PPS-dependent advantage in visual shape discrimination. It is beyond dispute that this dichotomy is not so strict (Milner & Goodale, 2008; Zachariou et al., 2015), and the dorsal pathway contains object representations that are, to some extent, independent from ventral ones (Freud, Culham, Plaut, & Behrmann, 2017; Freud, Ganel, et al., 2017; Freud, Plaut, & Behrmann, 2016; Quinlan & Culham, 2007; Wang, Li, Zhang, & Chen, 2016) and might contribute to perception. Additional candidate regions are a set of inferior parietal and premotor areas (Brozzoli et al., 2011; Fogassi et al., 1996; Graziano & Cooke, 2006; Rizzolatti et al., 1983) that are known to preferentially respond to stimuli presented in PPS. The latter neural network, which also includes the putamen (Graziano & Gross, 1993), contains a majority of neurons with bimodal (i.e., visual and tactile) receptive fields coding for PPS (Brozzoli et al., 2011; Fogassi et al., 1996), together with unimodal (visual) neurons. This network seems thus ideally suited to subserve the advantage in discriminating close versus far objects reported here.

Whereas future studies may tease apart the contribution of unisensory versus multisensory neurons in driving this advantage for PPS, here we disclose that depth per se, even when completely irrelevant for the situation at hand, helps to determine people’s visual perception of shapes, independently of physical size. In addition, we found that the sigmoidal performance curve, considered the fingerprint of the multisensory-defined boundary of PPS, can actually also be found for merely unimodal visual stimuli. The visual modality alone, therefore, can capture functional features of PPS that were previously thought to be exquisitely multisensory. Although we cannot state, at present, the extent to which visual and multisensory PPSs overlap, these findings open up new considerations in the ever-growing field of multisensory research: The convergence of multiple senses might not be a necessary feature to explain behavioral advantages in close space or even to probe PPS. We thus urge researchers conducting future studies to be depth aware, to better frame human visual abilities that are not homogeneously distributed in the three dimensions of the space around us.

## Supplementary Material

Supplementary material

Supplementary material

Supplementary material

Supplementary material

Supplementary material

## References

[bibr1-0956797618795679] Baayen R. H. Davidson D. J. Bates D. M. (2008). Mixed-effects modeling with crossed random effects for subjects and items. Journal of Memory and Language, 59, 390–412. doi:10.1016/j.jml.2007.12.005

[bibr2-0956797618795679] Brozzoli C. Ehrsson H. H. Farnè A. (2014). Multisensory representation of the space near the hand: From perception to action and interindividual interactions. The Neuroscientist, 20, 122–135. doi:10.1177/1073858413511153 24334708

[bibr3-0956797618795679] Brozzoli C. Gentile G. Ehrsson H. H. (2012). That’s near my hand! Parietal and premotor coding of hand-centered space contributes to localization and self-attribution of the hand. The Journal of Neuroscience, 32, 14573–14582. doi:10.1523/JNEUROSCI.2660-12.2012 23077043 PMC6621451

[bibr4-0956797618795679] Brozzoli C. Gentile G. Petkova V. I. Ehrsson H. H. (2011). fMRI adaptation reveals a cortical mechanism for the coding of space near the hand. The Journal of Neuroscience, 31, 9023–9031. doi:10.1523/JNEUROSCI.1172-11.2011 21677185 PMC6622953

[bibr5-0956797618795679] Canzoneri E. Magosso E. Serino A. (2012). Dynamic sounds capture the boundaries of peripersonal space representation in humans. PLOS ONE, 7(9), Article e44306. doi:10.1371/journal.pone.0044306 PMC3460958 23028516

[bibr6-0956797618795679] de Gonzaga Gawryszewski L. Riggio L. Rizzolatti G. Umiltá C (1987). Movements of attention in the three spatial dimensions and the meaning of “neutral” cues. Neuropsychologia, 25, 19–29. doi:10.1016/0028-3932(87)90040-6 3574647

[bibr7-0956797618795679] Ferri F. Tajadura-Jiménez A. Väljamäe A. Vastano R. Costantini M. (2015). Emotion-inducing approaching sounds shape the boundaries of multisensory peripersonal space. Neuropsychologia, 70, 468–475. doi:10.1016/j.neuropsychologia.2015.03.001 25744869

[bibr8-0956797618795679] Fogassi L. Gallese V. Fadiga L. Luppino G. Matelli M. Rizzolatti G. (1996). Coding of peripersonal space in inferior premotor cortex (area F4). Journal of Neurophysiology, 76, 141–157. 8836215 10.1152/jn.1996.76.1.141

[bibr9-0956797618795679] Freud E. Culham J. C. Plaut D. C. Behrmann M. (2017). The large-scale organization of shape processing in the ventral and dorsal pathways. eLife, 6, Article e27576. doi:10.7554/eLife.27576 PMC5659821 28980938

[bibr10-0956797618795679] Freud E. Ganel T. Shelef I. Hammer M. D. Avidan G. Behrmann M. (2017). Three-dimensional representations of objects in dorsal cortex are dissociable from those in ventral cortex. Cerebral Cortex, 27, 422–434. doi:10.1093/cercor/bhv229 26483400 PMC13100970

[bibr11-0956797618795679] Freud E. Plaut D. C. Behrmann M. (2016). ‘What’ is happening in the dorsal visual pathway. Trends in Cognitive Sciences, 20, 773–784. doi:10.1016/j.tics.2016.08.003 27615805

[bibr12-0956797618795679] Goodale M. A. Milner A. D. (1992). Separate visual pathways for perception and action. Trends in Neurosciences, 15, 20–25. doi:10.1016/0166-2236(92)90344-8 1374953

[bibr13-0956797618795679] Graziano M. S. A. Cooke D. F. (2006). Parieto-frontal interactions, personal space, and defensive behavior. Neuropsychologia, 44, 845–859. doi:10.1016/j.neuropsychologia.2005.09.009 16277998

[bibr14-0956797618795679] Graziano M. S. A. Gross C. G. (1993). A bimodal map of space: Somatosensory receptive fields in the macaque putamen with corresponding visual receptive fields. Experimental Brain Research, 97, 96–109. doi:10.1007/BF00228820 8131835

[bibr15-0956797618795679] Làdavas E. (2002). Functional and dynamic properties of visual peripersonal space. Trends in Cognitive Sciences, 6, 17–22. doi:10.1016/S1364-6613(00)01814-3 11849611

[bibr16-0956797618795679] Makin T. R. Holmes N. P. Brozzoli C. Farnè A. (2012). Keeping the world at hand: Rapid visuomotor processing for hand–object interactions. Experimental Brain Research, 219, 421–428. doi:10.1007/s00221-012-3089-5 22526949

[bibr17-0956797618795679] Makin T. R. Holmes N. P. Brozzoli C. Rossetti Y. Farnè A. (2009). Coding of visual space during motor preparation: Approaching objects rapidly modulate corticospinal excitability in hand-centered coordinates. The Journal of Neuroscience, 29, 11841–11851. doi:10.1523/JNEUROSCI.2955-09.2009 19776270 PMC6666640

[bibr18-0956797618795679] Matuschek H. Kliegl R. Vasishth S. Baayen H. Bates D. (2017). Balancing Type I error and power in linear mixed models. Journal of Memory and Language, 94, 305–315. doi:10.1016/j.jml.2017.01.001

[bibr19-0956797618795679] Milner A. D. Goodale M. A. (2008). Two visual systems re-viewed. Neuropsychologia, 46, 774–785. doi:10.1016/j.neuropsychologia.2007.10.005 18037456

[bibr20-0956797618795679] O’Connor D. A. Meade B. Carter O. Rossiter S. Hester R. (2014). Behavioral sensitivity to reward is reduced for far objects. Psychological Science, 25, 271–277. doi:10.1177/0956797613503663 24264939

[bibr21-0956797618795679] Plewan T. Rinkenauer G. (2017). Simple reaction time and size–distance integration in virtual 3D space. Psychological Research, 81, 653–663. doi:10.1007/s00426-016-0769-y 27030471

[bibr22-0956797618795679] Previc F. H. (1990). Functional specialization in the lower and upper visual fields in humans: Its ecological origins and neurophysiological implications. Behavioral & Brain Sciences, 13, 519–542. doi:10.1017/S0140525X00080018

[bibr23-0956797618795679] Quinlan D. J. Culham J. C. (2007). fMRI reveals a preference for near viewing in the human parieto-occipital cortex. NeuroImage, 36, 167–187. doi:10.1016/j.neuroimage.2007.02.029 17398117

[bibr24-0956797618795679] R Core Team. (2008). R: A language and environment for statistical computing. Retrieved from http://www.R-project.org

[bibr25-0956797618795679] Renner R. S. Velichkovsky B. M. Helmert J. R. (2013). The perception of egocentric distances in virtual environments—A review. ACM Computing Surveys, 46(2), Article 23. doi:10.1145/2543581.2543590

[bibr26-0956797618795679] Rizzolatti G. Fadiga L. Fogassi L. Gallese V. (1997). The space around us. Science, 277, 190–191. doi:10.1126/science.277.5323.190 9235632

[bibr27-0956797618795679] Rizzolatti G. Matelli M. Pavesi G. (1983). Deficits in attention and movement following the removal of postarcuate (area 6) and prearcuate (area 8) cortex in macaque monkeys. Brain, 106, 655–673. 6640275 10.1093/brain/106.3.655

[bibr28-0956797618795679] Sambo C. F. Forster B. Williams S. C. Iannetti G. D. (2012). To blink or not to blink: Fine cognitive tuning of the defensive peripersonal space. The Journal of Neuroscience, 32, 12921–12927. doi:10.1523/JNEUROSCI.0607-12.2012 22973016 PMC6703813

[bibr29-0956797618795679] Serino A. Canzoneri E. Avenanti A. (2011). Fronto-parietal areas necessary for a multisensory representation of peripersonal space in humans: An rTMS study. Journal of Cognitive Neuroscience, 23, 2956–2967. doi:10.1162/jocn_a_00006 21391768

[bibr30-0956797618795679] Teneggi C. Canzoneri E. di Pellegrino G. Serino A. (2013). Social modulation of peripersonal space boundaries. Current Biology, 23, 406–411. doi:10.1016/j.cub.2013.01.043 23394831

[bibr31-0956797618795679] van der Stoep N. Serino A. Farnè A. Di Luca M. Spence C (2016). Depth: The forgotten dimension in multisensory research. Multisensory Research, 29, 493–524. doi:10.1163/22134808-00002525

[bibr32-0956797618795679] Wang A. Li Y. Zhang M. Chen Q. (2016). The role of parieto-occipital junction in the interaction between dorsal and ventral streams in disparity-defined near and far space processing. PLOS ONE, 11(3), Article e0151838. doi:10.1371/journal.pone.0151838 PMC4801215 26999674

[bibr33-0956797618795679] Zachariou V. Nikas C. V. Safiullah Z. N. Behrmann M. Klatzky R. Ungerleider L. G. (2015). Common dorsal stream substrates for the mapping of surface texture to object parts and visual spatial processing. Journal of Cognitive Neuroscience, 27, 2442–2461. doi:10.1162/jocn_a_00871 26359538 PMC6632085

