# Supplementary material for: Mind the Depth: Visual Perception of Shapes Is Better in Peripersonal Space
Source: Psychol Sci. 2018 Oct 4;29(11):1868–77. doi: 10.1177/0956797618795679 (PMC6238160; doi:10.1177/0956797618795679)
Supplement: Supplementary material [file Exp_2_v0.html]

Distance effect - Exp 2, v1


# Distance effect - Exp 2, v1

#### *Elvio Blini INSERM U1028, ImpAct team, CRNL, and University of Lyon elvio.blini@gmail.com*

#### *13 September 2017*

# Experiment 2

This document includes data and analyses for the second experiment described in the companion paper.

We presented shapes - either cube or sphere - in the context of a Ponzo illusion (see below).

Ponzo illusion and time course of a typical trial.

Participants had to discriminate the presented shape by means of keypresses. Shapes were presented either illusorily close or far, but note that distance was irrelevant for the task at play.

We gave a time limit of 500 ms for the response. Also, responses faster than 100 ms were considered anticipations. A feedback was presented accordingly after response.

We focus on accuracy and reaction times (RTs) for correct answers that were provided within 100-500 ms.

For any request or inquiry don’t hesitate to contact: elvio.blini@gmail.com

## Preliminary setup

As a first step, ensure to clean the current environment to avoid conflicts. You can do it with `rm(list=ls())` (also ensure that modifications are saved for future use).

In order to run this script we need a few packages available on CRAN. You might need to install them first, e.g. by typing `install.packages("BayesFactor")` in the console.

```
#list packages
packages= c("ggplot2", "plyr", "BayesFactor", "lme4", "reshape", "gridExtra", "plot3D", "afex", "effsize")

#load them
lapply(packages, require, character.only= T)
```

```
## Warning: package 'ggplot2' was built under R version 3.4.4
```

```
## Warning: package 'effsize' was built under R version 3.4.4
```

Thanks to the function retrieved here, not displayed, the following hyperlink downloads the Rdata file:

That can be loaded then with:

```
load("Exp 2 data.RData")
```

Now all relevant variables are stored in the `data` data.frame, that you can navigate and explore with the usual commands, e.g. `str(data)`.

## Preprocessing

The factors Distance (Distanza) and Subject (subject\_nr) are to be converted into factors. Accuracy (correct) is numeric instead. The color of the shape was encoded as “Reward” because this experiment was the control condition of a larger one in which we linked each color to different outcomes; we only reported here a subset of subjects who performed this control condition as first task, to avoid carry-over effects.

```
data$Distanza= as.factor(data$Distanza)
data$subject_nr= as.factor(data$subject_nr)
data$correct= as.numeric(as.character(data$correct))

data$Color= as.factor(as.character(data$Reward))
levels(data$Color)= c("red", "blue", "green")
```

Practice trials were removed.

```
data= data[!is.na(data$time_Block),]
```

A response was considered **Valid** if it was correct and provided within 100 and 500 ms. We create a variable equals to 1 if this condition is met.

```
data$Valid= ifelse(data$correct == 1 & 
                   data$response_time>100 & 
                   data$response_time<500, 1, 0)
```

### Summarise accuracy

We summarise the percentage of correct responses, first for each subject.

We are interested, for this part, in the role of distance (depth of the shape).

```
acc.m.s= tapply(data$correct,
                list(data$subject_nr, data$Distanza),
                mean)
```

Then we obtain the grand average, sd, and sem.

```
#this averages across subjects (displayed)
(acc.m= apply(acc.m.s, 2, mean))
```

```
##     Close       Far 
## 0.9380208 0.9445313
```

```
#this calculates the standard deviation between subjects
(acc.sd= apply(acc.m.s, 2, sd))
```

```
##      Close        Far 
## 0.05320960 0.03980291
```

```
#this divides the sd by the square root of subjects' N
acc.sem= acc.sd/(sqrt(length(levels(data$subject_nr))))
```

What is the average percentage of trials omitted for both incorrect responses and slow RTs?

```
val.m.s= tapply(data$Valid,
                list(data$subject_nr, data$Distanza),
                mean)

#this averages across subjects (displayed)
(val.m= apply(val.m.s, 2, mean))
```

```
##     Close       Far 
## 0.8283854 0.8330729
```

```
#this calculates the standard deviation between subjects
(val.sd= apply(val.m.s, 2, sd))
```

```
##     Close       Far 
## 0.1402740 0.1244069
```

We now want to save the data frame as a separate object to run analyses over accuracy afterwards.

```
acc.an= data
```

Indeed, we now exclude wrong (plus too slow or too fast) responses to further assess RTs.

```
data= data[data$Valid==1,]
```

### Summarise RTs

We repeat the passages above to summarise reaction times.

```
rts.m.s= tapply(data$response_time,
                list(data$subject_nr, data$Distanza),
                mean)

#this averages across subjects (displayed)
(rts.m= apply(rts.m.s, 2, mean))
```

```
##    Close      Far 
## 391.0553 396.3116
```

```
#this calculates the standard deviation between subjects
rts.sd= apply(rts.m.s, 2, sd)

#this divides the sd by the square root of subjects' N
rts.sem= rts.sd/(sqrt(length(levels(data$subject_nr))))
```

## Analyses - Mixed Models

We now run statistics using (general) linear mixed-effects models.

The general strategy is to evaluate beforehand the random effects that increase model fitting, as to reach a parsimonious solution (i.e. supported by data). We create several different (nested) models and evaluate them against a simpler reference one through likelihood ratio tests (LRT). This holds for both random and fixed effects testing.

The simplest model to start with only includes the random intercept for Subjects (baseline level). We then start testing random slopes one by one, following this order:

1. Distance
2. Color of the shape
3. Shape (that is, correct answer)

Each random slope - that informs about variability in performance across levels of a factor, e.g. differences in experimental manipulations across subjects - will be retained in the model if the LRT is proven significant. Following evaluations will be made with reference models that include this slope. For example, if Distance improves model fit as random slope, Color will be evaluated against the model including it. As a second step we introduce interactions for all combinations of slopes proven significant (this is to respect marginality, and thus include high-order terms only together with their lower-order ones).

Fixed effects testing will use a similar (type 2) approach.

(To avoid verbosity, only the p value is shown).

### Accuracy

We start with the simplest model. Note that accuracy is binomial, thus we call for the general linear mixed effect regression (glmer) function and specify the family accordingly. I’m also asking for the “bobyqa” optimizer, which handles convergence problems very well.

```
mod0=glmer(correct ~ (1|subject_nr), data=acc.an, family=binomial, 
           control=glmerControl(optimizer="bobyqa"))
```

We create new models, each adding one piece of information at a time (random slopes, leftward part of the random formula specification).

```
#random slope for distance
mod0a=glmer(correct ~ (1+Distanza|subject_nr), data=acc.an, family=binomial, 
            control=glmerControl(optimizer="bobyqa"))

#LRT
anova(mod0, mod0a)$`Pr(>Chisq)`[2] #not really...
```

```
## [1] 0.0720216
```

Random slope for distance does not improve fit and would not therefore be retained in the final model.

```
#random slope for hand position
mod0b=glmer(correct ~ (1+Color|subject_nr), data=acc.an, family=binomial,
            control=glmerControl(optimizer="bobyqa"))
```

```
## Warning in checkConv(attr(opt, "derivs"), opt$par, ctrl = control
## $checkConv, : Model failed to converge with max|grad| = 0.00671207 (tol =
## 0.001, component 1)
```

```
#LRT  
anova(mod0, mod0b)$`Pr(>Chisq)`[2] #nope
```

```
## [1] 0.7401962
```

```
#slope for correct response - shape (Forma)
mod0c=glmer(correct ~ (1+Forma|subject_nr), data=acc.an, family=binomial,
            control=glmerControl(optimizer="bobyqa"))

anova(mod0, mod0c)$`Pr(>Chisq)`[2]
```

```
## [1] 0.5312556
```

No random slope improves model fit in this case.

```
mod.null= mod0
```

We can test the fixed effect of Distance.

```
#distance as fixed effect
mod1=glmer(correct ~ Distanza+ (1|subject_nr), data=acc.an, family=binomial,
           control=glmerControl(optimizer="bobyqa"))

#LRT
anova(mod.null, mod1)
```

```
## Data: acc.an
## Models:
## mod.null: correct ~ (1 | subject_nr)
## mod1: correct ~ Distanza + (1 | subject_nr)
##          Df    AIC    BIC  logLik deviance  Chisq Chi Df Pr(>Chisq)
## mod.null  2 3287.9 3301.8 -1641.9   3283.9                         
## mod1      3 3288.4 3309.2 -1641.2   3282.4 1.5193      1     0.2177
```

```
summary(mod1)
```

```
## Generalized linear mixed model fit by maximum likelihood (Laplace
##   Approximation) [glmerMod]
##  Family: binomial  ( logit )
## Formula: correct ~ Distanza + (1 | subject_nr)
##    Data: acc.an
## Control: glmerControl(optimizer = "bobyqa")
## 
##      AIC      BIC   logLik deviance df.resid 
##   3288.4   3309.2  -1641.2   3282.4     7677 
## 
## Scaled residuals: 
##     Min      1Q  Median      3Q     Max 
## -8.7511  0.1767  0.2074  0.2842  0.4704 
## 
## Random effects:
##  Groups     Name        Variance Std.Dev.
##  subject_nr (Intercept) 0.5537   0.7441  
## Number of obs: 7680, groups:  subject_nr, 32
## 
## Fixed effects:
##             Estimate Std. Error z value Pr(>|z|)    
## (Intercept)  2.94849    0.15188  19.413   <2e-16 ***
## DistanzaFar  0.12159    0.09768   1.245    0.213    
## ---
## Signif. codes:  0 '***' 0.001 '**' 0.01 '*' 0.05 '.' 0.1 ' ' 1
## 
## Correlation of Fixed Effects:
##             (Intr)
## DistanzaFar -0.304
```

Accuracy is not modulated by distance.

### Reaction times

We use the same selection procedure as above. For random effects we use restricted maximum likelihood (REML, that works well when fixed effects in the to-be-compared models are exactly the same). We’ll need to prevent the LRT to refit models. Here’s the simplest model.

```
rtmod0=lmer(response_time ~ (1|subject_nr), data=data, REML=T,
            control=lmerControl(optimizer="bobyqa"))
```

Then slopes are evaluated.

```
rtmod0a=lmer(response_time ~ (1+Distanza|subject_nr), data=data, REML=T, 
             control=lmerControl(optimizer="bobyqa"))
  
anova(rtmod0, rtmod0a, refit=F)$`Pr(>Chisq)`[2] #significant, keep it in
```

```
## [1] 0.003135521
```

```
rtmod0b=lmer(response_time ~ (1+Distanza+Color|subject_nr), data=data, REML=T, 
             control=lmerControl(optimizer="bobyqa"))

anova(rtmod0a, rtmod0b, refit=F)$`Pr(>Chisq)`[2] #nope
```

```
## [1] 0.8926407
```

```
rtmod0c=lmer(response_time ~ (1+Distanza+Forma|subject_nr), data=data, REML=T, 
             control=lmerControl(optimizer="bobyqa"))
  
anova(rtmod0a, rtmod0c, refit=F)$`Pr(>Chisq)`[2] #significant, keep it in
```

```
## [1] 5.296671e-10
```

We have two informative random slopes. Let’s assess their two-way interaction.

```
rtmod0d=lmer(response_time ~ (1+Distanza*Forma|subject_nr), data=data, REML=T, 
                 control=lmerControl(optimizer="bobyqa"))
    
anova(rtmod0c, rtmod0d, refit=F)$`Pr(>Chisq)`[2] #best
```

```
## [1] 3.984346e-08
```

So, the reference null model is the latter. For fixed effects we switch to maximum likelihood.

```
rtmod.null= rtmod0d
rtmod.null= update(rtmod.null, REML=F)
```

And then test for the fixed effect.

```
rtmod1=lmer(response_time ~ Distanza + (1+Distanza*Forma|subject_nr), data=data, REML=F,
              control=lmerControl(optimizer="bobyqa"))

anova(rtmod.null, rtmod1)
```

```
## Data: data
## Models:
## rtmod.null: response_time ~ (1 + Distanza * Forma | subject_nr)
## rtmod1: response_time ~ Distanza + (1 + Distanza * Forma | subject_nr)
##            Df   AIC   BIC logLik deviance  Chisq Chi Df Pr(>Chisq)  
## rtmod.null 12 67120 67201 -33548    67096                           
## rtmod1     13 67116 67204 -33545    67090 6.1145      1    0.01341 *
## ---
## Signif. codes:  0 '***' 0.001 '**' 0.01 '*' 0.05 '.' 0.1 ' ' 1
```

The main effect of distance is significant! So, let’s explore the model:

```
summary(rtmod1)
```

```
## Linear mixed model fit by maximum likelihood t-tests use Satterthwaite
##   approximations to degrees of freedom [lmerMod]
## Formula: response_time ~ Distanza + (1 + Distanza * Forma | subject_nr)
##    Data: data
## Control: lmerControl(optimizer = "bobyqa")
## 
##      AIC      BIC   logLik deviance df.resid 
##  67116.2  67204.0 -33545.1  67090.2     6367 
## 
## Scaled residuals: 
##     Min      1Q  Median      3Q     Max 
## -4.1376 -0.6784 -0.0582  0.6811  2.9050 
## 
## Random effects:
##  Groups     Name                   Variance Std.Dev. Corr             
##  subject_nr (Intercept)             243.1   15.59                     
##             DistanzaFar             130.2   11.41     0.19            
##             FormaSfera              184.3   13.57    -0.19  0.44      
##             DistanzaFar:FormaSfera  455.2   21.33    -0.05 -0.92 -0.67
##  Residual                          2096.2   45.78                     
## Number of obs: 6380, groups:  subject_nr, 32
## 
## Fixed effects:
##             Estimate Std. Error      df t value Pr(>|t|)    
## (Intercept)  389.950      2.787  30.560 139.940  < 2e-16 ***
## DistanzaFar    4.252      1.344  33.070   3.164  0.00333 ** 
## ---
## Signif. codes:  0 '***' 0.001 '**' 0.01 '*' 0.05 '.' 0.1 ' ' 1
## 
## Correlation of Fixed Effects:
##             (Intr)
## DistanzaFar -0.079
```

As you see there are no p values by default. There are many strategies to obtain (approximated) ones, here we’re happy with confidence intervals of the coefficient (that is, the adjustment with respect to the first level of Distance, 50 cm). The problem is that it takes a LOT of time on my machine (you are advised, :) ).

```
(ci= confint(rtmod1, method="boot", nsim=500, parm= 18))
```

This is what I got:

|  | 2.5 % | 97.5 % |
| --- | --- | --- |
| **Dist3** | 1.326732 | 7.095071 |

Since it is not straightforward to have an effect size index from mixed models, we switch to the good old Cohen’s d.

```
cohen.d(rts.m.s[,2], rts.m.s[,1], conf.level = 0.95,
                 hedges.correction = FALSE, paired= T)
```

```
## 
## Cohen's d
## 
## d estimate: 0.6823394 (medium)
## 95 percent confidence interval:
##       inf       sup 
## 0.1682601 1.1964188
```

## Robustness checks

Mixed models are a huge leap forward in statistical modelling (e.g. Baayen et al., 2008). For example, they can account for information gathered in every trial (opposed to data averaged for each condition), and allow an improved specification of random effects. But mixed models, also in light of the latter property, can be a dangerous minefield (Eager and Roy, 2017). For sure they expose researchers to dozens of (legitimate) degrees of freedom, which can potentially impact the false-positive rate of reported findings if no objective pipeline is followed.

Barr and colleagues (2013) proposed, in order to obviate to the problem, to simply add to the models all the random effects that are justified by the experimental design. Unfortunately, complicated matrices often result in convergence problems in the face of a negligible increase in the amount of information provided or even overfitting (Matuschek et al, 2017). In presence of convergence problems, estimated models simply cannot be interpreted, and present inflated type 2 errors (thus less power, Matuschek et al, 2017).

During the last years - because we often encountered convergence problems that could not be remediated by switching to different optimizers, especially when fitting binomial dependent variables (e.g. accuracy) - we established in the lab an objective, replicable pipeline and a set of criteria for selecting the most appropriate matrix of random effects (i.e. supported by the data). The pipeline is exposed at length in a recently published study (Blini et al., in press, Cortex). In this study we adopted the same procedure detailed in the paper, which was also pre-registered as such in that case. Adopting a common, objective strategy for our studies helps us be reassured that type 1 and 2 errors are balanced, on one hand, and that we are not subjects to manifold temptations arising from the several available forking paths.

To conclude, here we followed slavishly our self-imposed procedure (though recommended elsewhere, e.g. Matuschek et al., 2017).

However, in this section we decided to probe the results of mixed models with other inferential techniques (i.e. t-tests). The main intent is to confirm that results are genuine and not due to the specific procedure for selecting random effects employed here.

### T-test

We summarise the data (average for each subject and Distance condition).

```
DF= ddply(data, c("subject_nr", "Distanza"), summarise, dv= mean(response_time))
```

Then run the t-test:

```
t.test(x= DF$dv[DF$Distanza== "Far"], 
       y= DF$dv[DF$Distanza== "Close"], paired= TRUE)
```

```
## 
##  Paired t-test
## 
## data:  DF$dv[DF$Distanza == "Far"] and DF$dv[DF$Distanza == "Close"]
## t = 3.8599, df = 31, p-value = 0.0005384
## alternative hypothesis: true difference in means is not equal to 0
## 95 percent confidence interval:
##  2.478911 8.033540
## sample estimates:
## mean of the differences 
##                5.256225
```

Which confirms the effect of **Distance**. Actually, the p value drawn from mixed models appeared, in this case, much more conservative.

### Bayesian t-test

Second, we perform a Bayesian t-test. We use objective priors to avoid a few degrees of freedom (results partly depend on prior choice). This is possible thanks to the `BayesFactor` package. A Bayes Factor > 1 supports the alternative hypothesis, the null if < 1.

```
ttestBF(x= DF$dv[DF$Distanza== "Far"], 
            y= DF$dv[DF$Distanza== "Close"], paired= TRUE)
```

When compared against the model 

...

Enter one or more search terms in the box to filter the models in the table. If more than one term is included, matching will be performed with `or`. Special search terms are allowed:

| Code | Function | Example | What example does |
| --- | --- | --- | --- |
| + | Require this term in search results | +shape | Requires all models to include âshapeâ |
| - | Require this term NOT to appear in search results | -shape | Requires all models to exclude âshapeâ |
| # (with number) | Return results with certain number of terms | #2 | Requires all models to have two terms |
| @ (with :, ::, â¦) | Return results containing interactions of certain size | @:: | Requires all models to have a three-way interaction |
| < or > | Limits sizes of Bayes factors | >2 | Returns models whose Bayes factor is greater than 2 |

Click on a row in the Bayes factor table to make that model the denominator. Sort by clicking on the arrows in the column headers.

|  |  |  |
| --- | --- | --- |
| ...the model below... | ...is preferred by... |  |

From which we can see that the main effect of Distance is also supported by the Bayes Factor approach.

## Plots

```
Xs= melt(rts.m.s)
colnames(Xs)= c("Subject", "Distance", "RTs")
Xs$Distance= as.factor(Xs$Distance)
Xs$Color= ifelse(Xs$Distance== "Close", "light blue", "light green")
  
Xm= data.frame(Distance= c("Close", "Far"), 
                 RTs= rts.m, sem= rts.sem)
           
  #regular
  ggplot(Xm, aes(x= Distance, y= RTs)) +
    theme_bw() + theme(text= element_text(size=20, face="bold")) +
    geom_errorbar(data = Xm, aes(ymin= RTs-sem, ymax= RTs+sem), size= 1.5, width= .2, colour= "black") + 
    geom_point(size= 6, stroke= 2, shape= 21, color= "black", fill= c("blue", "green")) +
    ylab("RTs (ms)") +
    scale_y_continuous(limits= c(385, 400))
```

```
#lines 
ggplot(Xm, aes(x= Distance, y= RTs)) +
    theme_bw() + theme(text= element_text(size=20, face="bold")) +
    geom_line(data = Xs, aes(x= Distance, y= RTs, group= Subject), size= 1.1, color= "#007F7F", alpha= 0.5) +
    geom_errorbar(data = Xm, aes(ymin= RTs-sem, ymax= RTs+sem), size= 1.5, width= .2, colour= "black") + 
    geom_point(size= 6, stroke= 2, shape= 21, color= "black", fill= c("blue", "green")) +
    ylab("RTs (ms)")
```

```
  #CumDistr
  ggplot(data, aes(x= response_time)) + theme_bw() +
    theme(text= element_text(size=20, face="bold")) + xlim(250,500) +
    ylab("Cumulative Distribution") + xlab("RTs (ms)") +
    stat_ecdf(aes(group= Distanza, fill= Distanza), 
              geom="density", n=10, color= "black", size= 1.1) +
    scale_fill_manual(name="Distance", values= c("blue", "green")) + 
    theme(legend.key = element_blank()) + guides(fill= FALSE)
```

```
#boxplot+ deltas
p= ggplot(Xs, aes(x= Distance, y= RTs)) +
    theme_bw() + theme(text= element_text(size=20, face="bold")) +
    geom_boxplot(size= 1.2, outlier.size= 2, color= "black", fill= c("blue", "green")) +
    ylab("RTs (ms)") + xlab("Distance") 

deltas= with(Xs, sapply(levels(as.factor(Subject)), function(x) diff(RTs[Subject==x])))
deltas= data.frame(deltas)

d= ggplot(deltas, aes(x= factor(0), y= deltas)) +
    theme_bw() + theme(text= element_text(size=20, face="bold")) +
    geom_boxplot(size= 1.2, outlier.size= 2, color= "black", fill= c("#007F7F")) +
    ylab("Gain (ms)") + xlab("Distance effect") + scale_x_discrete(labels = "Far - Close")

(c= grid.arrange(p,d, layout_matrix = rbind(c(1,1, 2))))
```

```
## TableGrob (1 x 3) "arrange": 2 grobs
##   z     cells    name           grob
## 1 1 (1-1,1-2) arrange gtable[layout]
## 2 2 (1-1,3-3) arrange gtable[layout]
```
