# Supplementary material for: Mind the Depth: Visual Perception of Shapes Is Better in Peripersonal Space
Source: Psychol Sci. 2018 Oct 4;29(11):1868–77. doi: 10.1177/0956797618795679 (PMC6238160; doi:10.1177/0956797618795679)
Supplement: Supplementary material [file Exp_5_v0.html]

Distance effect - Exp 5, v1


# Distance effect - Exp 5, v1

#### *Elvio Blini INSERM U1028, ImpAct team, CRNL, and University of Lyon elvio.blini@gmail.com*

#### *13 September 2017*

# Experiment 5

This document includes data and analyses for the fifth experiment described in the companion paper.

We presented shapes - either cube or sphere - in a virtual environment. Participants had to discriminate the presented shape by means of keypresses. Shapes were presented at six different distances ranging from 50 cm to 300. Note that distance was irrelevant for the task at play.

We gave a time limit of 500 ms for the response. Also, responses faster than 100 ms were considered anticipations. A feedback was presented accordingly after response.

We focus on accuracy and reaction times (RTs) for correct answers that were provided within 100-500 ms.

For any request or inquiry don’t hesitate to contact: elvio.blini@gmail.com

## Preliminary setup

As a first step, ensure to clean the current environment to avoid conflicts. You can do it with `rm(list=ls())` (also ensure that modifications are saved for future use).

In order to run this script we need a few packages available on CRAN. You might need to install them first, e.g. by typing `install.packages("BayesFactor")` in the console.

```
#list packages
packages= c("ggplot2", "plyr", "lme4", "reshape", "gridExtra")

#load them
lapply(packages, require, character.only= T)
```

Thanks to the function retrieved here, not displayed, the following hyperlink downloads the Rdata file:

That can be loaded then with:

```
load("Exp 5 data.RData")
```

Now all relevant variables are stored in the `data` data.frame, that you can navigate and explore with the usual commands, e.g. `str(data)`.

## Preprocessing

The factors Distance (Dist) and Subject (SubjNb) are to be converted into factors.

```
data$Dist= as.factor(data$Dist)
data$SubjNb= as.factor(data$SubjNb)
```

Practice trials were removed.

```
data= data[data$Phase=="experiment",]
```

The Oculus Rift has a fixed latency of 20 ms that we subtract from subjects’ reaction times. Note that this is a constant value and does not affect statistics afterwards.

```
data$R_time= data$R_time - 20
```

A response was considered **Valid** if it was correct and provided within 100 and 500 ms. We create a variable equals to 1 if this condition is met.

```
data$Valid= ifelse(data$Accur. == 1 & 
                   data$R_time>100 & 
                   data$R_time<500, 1, 0)
```

### Summarise accuracy

We summarise the percentage of correct responses, first for each subject.

We are interested, for this part, in the role of distance (depth of the shape).

```
acc.m.s= tapply(data$Accur.,
                list(data$SubjNb, data$Dist),
                mean)
```

Then we obtain the grand average, sd, and sem.

```
#this averages across subjects (displayed)
(acc.m= apply(acc.m.s, 2, mean))
```

```
##       0.5         1       1.5         2       2.5         3 
## 0.9201389 0.9201389 0.9194444 0.8833333 0.8597222 0.8111111
```

```
#this calculates the standard deviation between subjects
acc.sd= apply(acc.m.s, 2, sd)

#this divides the sd by the square root of subjects' N
acc.sem= acc.sd/(sqrt(length(levels(data$SubjNb))))
```

We now want to save the data frame as a separate object to run analyses over accuracy afterwards.

```
acc.an= data
```

Indeed, we now exclude wrong (plus too slow or too fast) responses to further assess RTs.

```
data= data[data$Valid==1,]
```

### Summarise RTs

Now we centre RTs to the subject-specific mean. Negative values will thus indicate a relative advantage, whereas positive values slower responses.

```
#centre variables
for (i in 1:length(levels(data$SubjNb))){
  data$R_time[data$SubjNb== i]= data$R_time[data$SubjNb== i]- 
                              mean(data$R_time[data$SubjNb== i])
}
```

We repeat the passages above to summarise reaction times.

```
rts.m.s= tapply(data$R_time,
                list(data$SubjNb, data$Dist),
                mean)

#this averages across subjects (displayed)
(rts.m= apply(rts.m.s, 2, mean))
```

```
##        0.5          1        1.5          2        2.5          3 
##  -9.163308 -11.861315  -6.222044   5.787019   8.967053  19.275276
```

```
#this calculates the standard deviation between subjects
rts.sd= apply(rts.m.s, 2, sd)

#this divides the sd by the square root of subjects' N
rts.sem= rts.sd/(sqrt(length(levels(data$SubjNb))))
```

### Fit evaluation

This is a custom formula for RMSE:

```
#formula for RMSE
rmse <- function(error) {
  sqrt(mean(error^2))}
```

And this is a custom plot to summarise various models fit:

```
plot.fit= function(x, y, fit.model, main, xlim=NULL, plotSE=F, odds= F){
  
  pnts= tapply(y, x, mean)
  
  if (! is.null(fit.model)){
    main=paste0(main, ", AIC= ", round(AIC(fit.model), 2), ", RMSE= ", round(rmse(resid(fit.model)), 2))
    y.predicted={}
    y.predicted= predict(fit.model, 
                         newdata= list(x= seq(min(x), max(x), length=1000)))
    
    SE= tryCatch(summary(fit.model)$sigma, error= function(dummy){})
    if (! is.numeric(SE))(SE<- 0.00001)
    
    if (plotSE){
      if (is.null(xlim))
        (xlim= c(min(range(y.predicted), min(pnts))-SE, max(range(y.predicted), max(pnts))+ SE))
    } else {
      xlim= c(min(range(y.predicted), min(pnts)), max(range(y.predicted), max(pnts)))
    }
  } else {xlim= c(min(pnts), max(pnts))}
  
  if(odds)(xlab= "Logit") else (xlab= "Coefficient (ms)")
  
  plot(as.numeric(as.character(names(pnts))) ~ pnts, 
       cex.main=1.5, cex.axis=1.5, cex.lab=1.5, pch= 19, font= 2, font.lab= 2, 
       main= main, ylab= "Depth (cm)", xlab= xlab,
       ylim=c(0, 320), 
       xlim= xlim)
  
  if (plotSE)(
    polygon(y = c(seq(min(x), max(x), length= 1000),
                  seq(max(x), min(x), length= 1000)),
            x= c(y.predicted - SE, y.predicted[length(y.predicted):1] +SE),
            col= adjustcolor("lightgray", alpha.f= 0.2), border= NA))
  
  
  lines(x= c(0,0), y= range(x)+c(-10, 10), lty=2, col="darkgray")
  
  #fixation
  lines(x= xlim, y= c(175,175), lty=2, col="darkgray")
  text(xlim[1], y= 170, "Fixation", pos= 4)
  
  if (! is.null(fit.model)){
    lines(y = seq(min(x), max(x), length=1000), x= y.predicted, lwd= 2)
  
  if (plotSE){
    if (SE== 0.00001)
      (text(x = xlim[1], y= 5, labels= "SE could not be obtained", pos= 4))}

}}
```

## Mixed models

We now fit two different mixed models with a random slope. Each participant will thus have its own trend to fit, beside the group average.

For accuracy:

```
mod.acc=glmer(Accur. ~ Dist + (1+Dist|SubjNb), data=acc.an, family= binomial,
           control=glmerControl(optimizer="bobyqa"))
```

We extract random slopes. Values are initially coded as adjustments with respect to the first value (intercept), so we need to reshape these values to obtain absolute indices:

```
RS.acc.s= coef(mod.acc)$SubjNb
RS.acc.s[,2:6]= RS.acc.s[,1]+RS.acc.s[,2:6]
colnames(RS.acc.s)= as.character(seq(50, 300, by= 50))
```

The group average:

```
(RS.acc.m= colMeans(RS.acc.s))
```

```
##       50      100      150      200      250      300 
## 2.532915 2.503224 2.504583 2.102547 1.863762 1.517337
```

Results are in terms of odds to produce a correct response. Now for RTs.

```
mod.rts=lmer(R_time ~ Dist + (1+Dist|SubjNb), data=data, 
                      REML=F, control=lmerControl(optimizer="bobyqa"))
RS.rts.s= coef(mod.rts)$SubjNb
RS.rts.s[,2:6]= RS.rts.s[,1]+RS.rts.s[,2:6]
colnames(RS.rts.s)= as.character(seq(50, 300, by= 50))
(RS.rts.m= colMeans(RS.rts.s))
```

```
##         50        100        150        200        250        300 
##  -9.349406 -11.956616  -6.073964   5.932011   9.042909  18.721719
```

## Fit

For all computations the independent variable will be depth:

```
x= seq(50, 300, by= 50)
```

Then the strategy consists of four steps:

1. Provide a first guess of what the parameters could be (very important);
2. Optimize the guess to help the following procedures (via the `optim` function);
3. Fit models through the `nls` function;
4. Depict results and summary statistics.

We first assess the group means for RTs and accuracy, then single subjects.

We can take advantage of the fact that formulas are all the same, and declare something in advance. For example, to optimize our initial guess we need custom functions to be minimized accordingly.

```
# log
log.min.fn= function(data, par) {
  with(data, 
       sum((par[[1]] + par[[2]]*log(x)  - y )^2))
  
}

# exp 
exp.min.fn=function(data, par) {
  with(data, 
       sum((par[[1]] + par[[2]]*exp(x/100) - y )^2))
  
}

# sigmoidal
sig.min.fn=function(data, par) {
  with(data, 
       sum((par[[1]] + ((par[[2]] - par[[1]])/(1 + exp(par[[3]] * (x - par[[4]]))))  - y )^2))
  
}
```

The linear trend needs no optimization as it is straigthforward. As you can see the sigmoidal curve (general form) needs 2 additional parameters in order to be described. This is why, beside the RMSE, we also ask for the Akaike Information Criterion, to control for potential overfitting of the data.

We can also declare a general fitting function:

```
my_fitting_function= function(what){
  
  #initial error, to minimize across cycles
  Error= Inf
  
  #choose appropriate guess matrix
  GUESS= list(log= GUESS.log, exp= GUESS.exp, sig= GUESS.sig)[what][[1]]
  
  for (i in 1:nrow(GUESS)){
  
  #choose guess
  starting= as.list(GUESS[i,])
  
  #choose minimize function, then optimize
  minimize.function= list(log= log.min.fn, exp= exp.min.fn, sig= sig.min.fn)[what][[1]]
  
  optimized.values= optim(par= starting, minimize.function, data=data.frame(x= x, y= y),
                          method= "SANN")$par
  
  #fit the relevant curve
  fit= {}
  if(what=="log"){
      fit= tryCatch(nls(y ~ a + b*log(x), start= optimized.values,
               control = list(maxiter = 10^4, warnOnly= T), 
               algorithm = "port"), error= function(dummy){})} 
  
  if(what=="exp"){
      fit= tryCatch(nls(y ~ a + b*exp(x/100), start= optimized.values,
             control = list(maxiter = 10^4, warnOnly= T), 
             algorithm = "port"), error= function(dummy){})}
  
  if(what=="sig"){
      fit= tryCatch(nls(y ~ a + ((b - a)/(1 + exp(c * (x - d)))), 
                start= optimized.values,
                control = list(maxiter = 10^4, warnOnly= T), 
                algorithm = "port"), error= function(dummy){})}
  
  #check if model has been estimated, then if Error is less than previous model.
  #retain model if successful
  if(!is.null(fit)) {
    if(rmse(resid(fit)) < Error) {Error= rmse(resid(fit)); final.fit= fit}
    if(fit$convInfo$isConv) break;}
  
  }

  return(final.fit)
}
```

If successful this function takes as only arguments the function that we want to fit. It automatically search for x and y in the environment, as well as our initial guess and the function to minimize residuals (passed to `optim`).

A few things to notice: 1. Everything is wrapped around a loop such that each cycle tests a different provisional guess. The cycle breaks when the model is successfully estimated. 2. The main function, `nls`, is wrapped around `TryCatch` to avoid convergence problems to break the loop. Note that this does not necessarily get rid of convergence problems… Visual inspection of predicted lines will be the most important criterion after this first step. Furthermore, note that this is designed for sequential testing (i.e. it takes objects from the environment that change at every step - e.g. `y` is overwritten from RTs to accuracy!)

### RTs

We declare in advance our guesses for initial parameters.

```
GUESS.log= expand.grid(a= seq(-80, -50, 5), b= seq(5, 25, 5))

GUESS.exp= expand.grid(a= seq(-50, -5, 5), b= seq(1, 10, 2))

GUESS.sig= expand.grid(a= seq(-20, -5, 5), b= seq(10, 150, 30),
                       c= seq(-0.01, -0.4, -0.05), d= seq(100, 200, 50))
```

The dependent variable is:

```
y= RS.rts.m
```

Linear fit is very basic (same as `lm`).

```
# linear fit
lin.fit= nls(y ~ a+b*x, start= list(a= -30, b= -10),
                control = list(maxiter = 500, warnOnly=T))
```

Trickier, the logaritmic trend:

```
log.fit= my_fitting_function("log")
```

Now fit an exponential curve:

```
exp.fit= my_fitting_function("exp")
```

And finally the sigmoid trend:

```
sig.fit= my_fitting_function("sig")
```

Now a graphical depiction of all results:

```
par(mfrow=c(2,2))
plot.fit(x, y, lin.fit, "Linear")
plot.fit(x, y, log.fit, "Log")
plot.fit(x, y, exp.fit, "Exp")
plot.fit(x, y, sig.fit, "Sigmoid")
```

The sigmoid curve appears the best one for both criteria (RMSE and AIC)!

A fancy plot:

```
DF= data.frame(x= x, y= y, sem= apply(RS.rts.s, 2, sd)/sqrt(length(levels(data$SubjNb))))
#only for prediction purposes
extra= data.frame(x= c(0, 350, 400), y= c(NA, NA, NA), sem= c(NA, NA, NA))

coef.plot= function(x)(coef(sig.fit)[1] + ((coef(sig.fit)[2] - coef(sig.fit)[1])/(1 + exp(coef(sig.fit)[3] * (x - coef(sig.fit)[4])))))

ggplot(DF, aes(x= x, y= y)) + scale_x_continuous(limits= c(0, 400), 
                                                 breaks= seq(50, 300, by= 50), 
                                                 labels=c("D1", "D2", "D3", "D4", "D5", "D6")) +
  theme_bw() + theme(text= element_text(size=20, face="bold")) +
  ylab("RTs Advantage (ms)") + xlab("Distance") +
  stat_function(fun= coef.plot, color= "#007F7F", size= 1.2) +
  geom_errorbar(aes(ymin= y-sem, ymax= y+sem), size= 1.5, width= .2, colour= "black") +
  geom_point(size= 5, stroke= 2, shape= 21, color= "black", fill= "blue")
```

Now we want to identify measures indexing the **limits** of PPS. We have framed RTs in terms of *relative advantage* with respect to the subject-specific mean: the estimated point in which the advantage is equal to 0 could be taken as a proxy for the PPS limit. We will need the `nls2` package.

```
predict(sig.fit, newdata= data.frame(x= seq(183, 185, by= 1)))
```

```
## [1] -0.12941990  0.04591157  0.22229769
```

Thus 184 cm. Another way is to calculate the inflection point of the sigmoid. We’ll take the maximum of the derivative.

```
par(mfrow=c(2,1))

y= coef.plot(50:300)
plot(y= y, x= 50:300, type= "l", lwd= 3)
abline(v= 209, col= "dark gray", lty= 2, lwd= 3)

y= c(NA, diff(y))
plot(x= 50:300, y= y, type= "l", lwd= 3)
abline(v= 209, col= "dark gray", lty= 2, lwd= 3)
```

```
seq(50, 300)[y== max(y, na.rm= T)]
```

```
## [1]  NA 209
```

The inflection point thus is around 209 cm.

### Accuracy

Our guesses:

```
GUESS.log= expand.grid(a= seq(1, 10, 1), b= seq(-10, 0, 1))

GUESS.exp= expand.grid(a= seq(6, 8, 0.5), b= seq(-0.01, -1, -0.05))

GUESS.sig= expand.grid(a= seq(13, 17, 1), b= seq(-12, 8, 1),
                       c= seq(-0.01, -0.4, -0.05), d= seq(50, 100, 10))
```

The dependent variable is:

```
y= RS.acc.m
```

Linear fit is very basic.

```
# linear fit
lin.fit= nls(y ~ a+b*x, start= list(a= -30, b= -10),
                control = list(maxiter = 500, warnOnly=T))
```

The logaritmic trend:

```
log.fit= my_fitting_function("log")
```

Now, this is hard, fit an exponential curve:

```
exp.fit= my_fitting_function("exp")
```

And finally the sigmoid trend:

```
sig.fit= my_fitting_function("sig")
```

Now a graphical depiction of all results:

```
par(mfrow=c(2,2))
plot.fit(x, y, lin.fit, "Linear", odds = T)
plot.fit(x, y, log.fit, "Log", odds = T)
plot.fit(x, y, exp.fit, "Exp", odds = T)
plot.fit(x, y, sig.fit, "Sigmoid", odds = T)
```

The sigmoid curve appears the best one for both criteria!

A fancy plot:

```
DF= data.frame(x= x, y= y, sem= apply(RS.acc.s, 2, sd)/sqrt(length(levels(data$SubjNb))))
#only for prediction purposes
extra= data.frame(x= c(0, 350, 400), y= c(NA, NA, NA), sem= c(NA, NA, NA))

coef.plot= function(x)(coef(sig.fit)[1] + ((coef(sig.fit)[2] - coef(sig.fit)[1])/(1 +     exp(coef(sig.fit)[3] * (x - coef(sig.fit)[4])))))

ggplot(DF, aes(x= x, y= y)) + scale_x_continuous(limits= c(0, 400), 
                                                 breaks= seq(50, 300, by= 50),
                                                 labels=c("D1", "D2", "D3", "D4", "D5", "D6")) +
  theme_bw() + theme(text= element_text(size=20, face="bold")) +
  ylab("Odds") + xlab("Distance") +
  stat_function(fun= coef.plot, color= "#007F7F", size= 1.2) +
  geom_errorbar(aes(ymin= y-sem, ymax= y+sem), size= 1.5, width= .2, colour= "black") +
  geom_point(size= 5, stroke= 2, shape= 21, color= "black", fill= c("blue"))
```

The inflection point:

```
par(mfrow=c(2,1))

y= coef.plot(50:300)
plot(y= y, x= 50:300, type= "l", lwd= 3)
abline(v= 233, col= "dark gray", lty= 2, lwd= 3)

y= c(NA, diff(y))
plot(x= 50:300, y= y, type= "l", lwd= 3)
abline(v= 233, col= "dark gray", lty= 2, lwd= 3)
```

```
seq(50, 300)[y== min(y, na.rm= T)]
```

```
## [1]  NA 233
```

## Single subjects level

Now we can explore each trend at the subject level, to see the proportion of subjects whose performance is better represented by a sigmoid.

## RTs

We provide wider guesses to account for inter-individual variability.

```
GUESS.log= expand.grid(a= seq(-100, -30, 5), b= seq(1, 45, 5))

GUESS.exp= expand.grid(a= seq(-70, -1, 5), b= seq(1, 20, 2))

GUESS.sig= expand.grid(a= seq(-30.1, 0.1, 10), b= seq(10, 190, 60),
                       c= seq(-0.01, -0.41, -0.05), d= seq(100, 300, 50))
```

We wrap everything in a loop. Plots will be saved in a subfolder, here we only retain RMSE and AIC.

```
#store results
results.rts= {}

for (i in 1: length(levels(data$SubjNb))){
  
  y= unlist(RS.rts.s[i,])
  
  #linear fit
  lin.fit= nls(y ~ a+b*x, start= list(a= -30, b= -10),
                control = list(maxiter = 500, warnOnly=T))
  #log
  log.fit= my_fitting_function("log")
  
  #exp
  exp.fit= my_fitting_function("exp")
  
  #sig
  sig.fit= my_fitting_function("sig")

  #plot
  par(mfrow=c(2,2))
  plot.fit(x, y, lin.fit, "Linear")
  plot.fit(x, y, log.fit, "Log")
  plot.fit(x, y, exp.fit, "Exp")
  plot.fit(x, y, sig.fit, "Sigmoid")
  
  what= c("lin", "log", "exp", "sigm")
  #minimum RMSE?
  minRMSE= what[which.min(c(rmse(resid(lin.fit)),
            rmse(resid(log.fit)),
            rmse(resid(exp.fit)),
            rmse(resid(sig.fit))))]
  #minimum AIC?
  minAIC= what[which.min(c(AIC(lin.fit),
            AIC(log.fit),
            AIC(exp.fit),
            AIC(sig.fit)))]

  results.rts= rbind(results.rts,
                 cbind(RMSE= minRMSE, AIC= minAIC))  
  }
```

Now we can summarise results. First for RMSE:

```
table(results.rts[,1])
```

```
## 
## sigm 
##   20
```

All subjects! Let’s look at the AIC:

```
table(results.rts[,2])
```

```
## 
##  exp sigm 
##    9   11
```

Eleven over 20 subjects! 55% for the sigmoidal and 45% for exponential curve, a close call.

## Accuracy

We provide wider guesses to account for inter-individual variability.

```
GUESS.log= expand.grid(a= seq(1, 20, 1), b= seq(-20, 0, 1))

GUESS.exp= expand.grid(a= seq(4, 10, 0.5), b= seq(-0.01, -2, -0.05))

GUESS.sig= expand.grid(a= seq(2, 26, 8), b= seq(-10.1, 5.1, 5),
                       c= seq(-0.01, -0.51, -0.1), d= seq(25, 275, 50))
```

We wrap everything in a loop. Plots will be saved in a subfolder, here we only retain RMSE and AIC.

```
#store results
results.acc= {}

for (i in 1: length(levels(data$SubjNb))){
  
  y= unlist(RS.acc.s[i,])
  
  #linear fit
  lin.fit= nls(y ~ a+b*x, start= list(a= -30, b= -10),
                control = list(maxiter = 500000, warnOnly=T))
  #log
  log.fit= my_fitting_function("log")
  
  #exp
  exp.fit= my_fitting_function("exp")
  
  #sig
  sig.fit= my_fitting_function("sig")

  #plot
  par(mfrow=c(2,2))
  plot.fit(x, y, lin.fit, "Linear", odds= T)
  plot.fit(x, y, log.fit, "Log", odds= T)
  plot.fit(x, y, exp.fit, "Exp", odds= T)
  plot.fit(x, y, sig.fit, "Sigmoid", odds= T)
  
  what= c("lin", "log", "exp", "sigm")
  #minimum RMSE?
  minRMSE= what[which.min(c(rmse(resid(lin.fit)),
            rmse(resid(log.fit)),
            rmse(resid(exp.fit)),
            rmse(resid(sig.fit))))]
  #minimum AIC?
  minAIC= what[which.min(c(AIC(lin.fit),
            AIC(log.fit),
            AIC(exp.fit),
            AIC(sig.fit)))]

  results.acc= rbind(results.acc,
                 cbind(RMSE= minRMSE, AIC= minAIC))  
  }
```

Now we can summarise results. First for RMSE:

```
table(results.acc[,1])
```

```
## 
## sigm 
##   20
```

The sigmoidal trend is preferred for all subjects but:

```
table(results.acc[,2])
```

```
## 
##  exp  log sigm 
##   11    1    8
```

The exponential curve takes the lead back when using the AIC, although slightly.
